# Supplementary material for: Conditioned respiratory threat in the subdivisions of the human periaqueductal gray
Source: eLife. 2016 Feb 27;5:e12047. doi: 10.7554/eLife.12047 (PMC4821794; doi:10.7554/eLife.12047)
Supplement: Figure 5—source data 1. — Values derived from cluster-based analysis. The most significant maximum is listed for each anatomical location. Co-ordinates are in mm in standard space of MNI (1 mm3). x, distance right (+) or left (-) of the mid saggital line; y, distance anterior (+) or posterior (-) from a vertical plane through the anterior commissure; z, distance above (+) or below (-) the intercommisurial plane. Abbreviations: VPL, ventroposterolateral nucleus of the thalamus. DOI: http://dx.doi.org/10.7554/eLife.12047.010 [file elife-12047-fig5-data1.docx]

| Locations of signal maxima in response to inspiratory resistance | | | | | | | | |
| --- | --- | --- | --- | --- | --- | --- | --- | --- |
| Region | left | | | | right | | | |
|  | x | y | z | max Z  score | x | y | z | max Z score |
| *Activations* | | | | | | | | |
| Motor cortex | -58 | 4 | 70 | 6.73 | 59 | 2 | 40 | 6.55 |
| Supplementary motor cortex | -3 | -1 | 60 | 6.73 | 1 | -4 | 70 | 4.82 |
| Putamen | -25 | -10 | 3 | 6.44 | 27 | -2 | -2 | 4.09 |
| Sensory cortex | -59 | -17 | 34 | 5.88 | 62 | -13 | 28 | 5.41 |
| Cingulate cortex | -6 | 13 | 35 | 3.59 | 9 | 13 | 37 | 4.32 |
| Paracingulate cortex | -7 | 7 | 44 | 3.33 | 3 | 18 | 49 | 5.46 |
| Operculum | -49 | 0 | 3 | 5.06 | 59 | -1 | 10 | 4.79 |
| Medulla | -2 | -47 | -64 | 3.50 | 7 | -45 | -64 | 3.58 |
| Middle insula | -37 | 2 | 5 | 5.21 | 39 | 2 | 6 | 4.96 |
| Caudate nucleus | -13 | -3 | 22 | 3.69 | 15 | 0 | 18 | 4.0 |
| VPL (thalamus) | -12 | -24 | 2 | 4.64 | 14 | -20 | -4 | 4.43 |
| Subthalamic nucleus | -2 | -20 | -3 | 5.11 | 13 | -19 | -4 | 3.55 |
| *Deactivations* | | | | | | | | |
| Hippocampus | -28 | -14 | -16 | 4.56 | 21 | -20 | -15 | 4.32 |
| Amygdala | -21 | -6 | -12 | 5.21 | 20 | -2 | -19 | 4.17 |
| Cerebellum | -7 | -48 | -48 | 3.76 | 8 | -49 | -45 | 3.77 |
