# Supplementary material for: Conditioned respiratory threat in the subdivisions of the human periaqueductal gray
Source: eLife. 2016 Feb 27;5:e12047. doi: 10.7554/eLife.12047 (PMC4821794; doi:10.7554/eLife.12047)
Supplement: Figure 5—source data 2. — Values derived from cluster-based analysis. The most significant maximum is listed for each anatomical location. Co-ordinates are in mm in standard space of MNI (1 mm3). x, distance right (+) or left (-) of the mid saggital line; y, distance anterior (+) or posterior (-) from a vertical plane through the anterior commissure; z, distance above (+) or below(-) the intercommisurial plane. DOI: http://dx.doi.org/10.7554/eLife.12047.011 [file elife-12047-fig5-data2.docx]

| Locations of signal maxima during certain anticipation of inspiratory resistance | | | | | | | | |
| --- | --- | --- | --- | --- | --- | --- | --- | --- |
| Region | left | | | | right | | | |
|  | x | y | z | max Z  score | x | y | z | max Z score |
| *Activations* | | | | | | | | |
| Motor cortex | -48 | -6 | 47 | 3.42 | 49 | 2 | 50 | 4.43 |
| Supplementary motor cortex | -1 | -1 | 72 | 4.98 | 6 | 6 | 66 | 3.97 |
| Sensory cortex | -58 | -18 | 34 | 3.44 | 3 | -19 | 27 | 4.43 |
| Cingulate cortex | -1 | -23 | 30 | 3.95 | 3 | -19 | 27 | 4.43 |
| Paracingulate cortex | -1 | 14 | 43 | 4.60 | 1 | 14 | 42 | 3.93 |
| Operculum | -45 | 4 | 0 | 2.94 | 49 | 2 | 4 | 4.60 |
| Medulla |  |  |  |  | 5 | -40 | -60 | 3.25 |
| Middle insula | -38 | 2 | 1 | 3.51 | 42 | 5 | 3 | 3.81 |
| *Deactivations* | | | | | | | | |
| Hippocampus | -34 | -23 | -16 | 4.07 | 26 | -11 | -16 | 3.93 |
| Amygdala | -19 | -6 | -13 | 4.24 | 16 | -2 | -1 | 4.07 |
| Posterior insula | -36 | -16 | 18 | 2.98 | 36 | -18 | 19 | 3.30 |
| Cerebellum |  |  |  |  | 7 | -52 | -51 | 3.64 |
| Locations of signal maxima during uncertain anticipation of inspiratory resistance | | | | | | | | |
| Region | left | | | | right | | | |
|  | x | y | z | max Z  score | x | y | z | max Z score |
| *Activations* | | | | | | | | |
| Motor cortex | -47 | -6 | 47 | 3.91 | 55 | 9 | 41 | 3.62 |
| Supplementary motor cortex | -1 | -2 | 71 | 4.53 | 6 | 0 | 71 | 4.23 |
| Sensory cortex | -55 | -18 | 27 | 3.64 | 50 | -15 | 25 | 3.93 |
| Anterior cingulate cortex | -7 | 15 | 39 | 3.73 | 9 | 15 | 40 | 4.01 |
| Posterior cingulate cortex | -1 | -21 | 32 | 3.61 | 4 | -19 | 27 | 3.81 |
| Operculum | -43 | -4 | 13 | 3.28 | 46 | 4 | 3 | 3.39 |
| Middle insula | -39 | 3 | 3 | 3.53 | 42 | 5 | 2 | 3.52 |
| *Deactivations* | | | | | | | | |
| Hippocampus | -33 | -23 | -12 | 3.36 | 22 | -15 | -14 | 3.31 |
| Amygdala | -20 | -7 | -12 | 3.63 | 21 | -12 | -12 | 2.90 |
| Posterior insula | -35 | -22 | 20 | 3.44 | 37 | -18 | 18 | 3.36 |
| Cerebellum |  |  |  |  | 5 | -56 | -46 | 3.77 |
