# Supplementary material for: Conditioned respiratory threat in the subdivisions of the human periaqueductal gray
Source: eLife. 2016 Feb 27;5:e12047. doi: 10.7554/eLife.12047 (PMC4821794; doi:10.7554/eLife.12047)
Supplement: Figure 5—source data 3. — Values derived from cluster-based analysis. The most significant maximum is listed for each anatomical location. Co-ordinates are in mm in standard space of MNI (1 mm3). x, distance right (+) or left (-) of the mid saggital line; y, distance anterior (+) or posterior (-) from a vertical plane through the anterior commissure; z, distance above (+) or below (-) the intercommisurial plane. Abbreviations: VPL, ventroposterolateral nucleus of the thalamus. DOI: http://dx.doi.org/10.7554/eLife.12047.012 [file elife-12047-fig5-data3.docx]

| Locations of signal maxima during finger opposition | | | | | | | | |
| --- | --- | --- | --- | --- | --- | --- | --- | --- |
| Region | left | | | | right | | | |
|  | x | y | z | max Z  score | x | y | z | max Z score |
| *Activations* | | | | | | | | |
| Motor cortex | -44 | -16 | 52 | 6.07 | 52 | -3 | 47 | 3.60 |
| Supplementary motor cortex | -1 | -5 | 57 | 5.21 | 5 | 0 | 58 | 4.72 |
| Putamen | -31 | -10 | -1 | 6.63 | 24 | 7 | 2 | 4.83 |
| Sensory cortex | -57 | -23 | 23 | 4.11 | 58 | -17 | 25 | 3.46 |
| Cingulate cortex | -5 | 11 | 35 | 4.29 | 5 | -7 | 37 | 3.88 |
| Paracingulate cortex | -4 | 12 | 42 | 4.86 | 12 | 8 | 41 | 3.96 |
| Operculum | -43 | 0 | 11 | 6.89 | 49 | 1 | 7 | 6.21 |
| Medulla | -1 | -47 | -61 | 3.30 | 5 | -49 | -60 | 4.60 |
| Caudate nucleus | -10 | 0 | 16 | 3.52 | 10 | 4 | 12 | 3.40 |
| VPL (thalamus) | -15 | -24 | 5 | 6.54 | 12 | -18 | 12 | 4.00 |
| *Deactivations* | | | | | | | | |
| Hippocampus |  |  |  |  | 27 | -20 | -13 | 3.66 |
| Motor cortex | -45 | -11 | 37 | 4.48 | 58 | -4 | 35 | 4.04 |
| Posterior insula |  |  |  |  | 36 | -5 | 15 | 4.34 |
